# Association between social capital and self-rated health among community-dwelling older adults

**DOI:** 10.3389/fpubh.2022.916485

**Published:** 2022-09-08

**Authors:** Zhongliang Bai, Jing Yang, Zijing Wang, Wenwen Cao, Chenglin Cao, Zhi Hu, Ren Chen

**Affiliations:** ^1^Department of Health Services Management, School of Health Services Management, Anhui Medical University, Hefei, China; ^2^Department of Epidemiology and Biostatistics, School of Public Health, Anhui Medical University, Hefei, China; ^3^Educational Institute of Behavioral Medicine, Jining Medical University, Jining, China; ^4^Affiliated Suzhou Hospital of Anhui Medical University, Suzhou, China

**Keywords:** social capital, self-rated health, older people, health aging, China

## Abstract

**Background:**

It is less known about whether the association of social capital with self-rated health (SRH) varies by the presence of health conditions and how social capital, together with other variables, is linked to self-rated health in later life.

**Objectives:**

This article aimed to explore the association of social capital with self-rated health and to examine whether the association varies among older people with and without health conditions, with a special focus on how social capital and relevant factors have an effect on self-rated health among community-dwelling older adults.

**Methods:**

Cross-sectional data were obtained from a survey that commenced from July to September 2017 in Anhui Province. Data on socio-demographic information, social capital (six dimensions), and self-rated health were analyzed. Binary logistic regression and classification and regression tree (CART) models were used to estimate the association.

**Results:**

Based on the findings, we found that social capital regarding less social support (adjusted odds ratio (*AOR*) = 1.57, 95% *CI*: 1.21–2.04), and less reciprocity (*AOR* = 1.73, 95% *CI*: 1.29–2.31) were associated with self-rated health among general older adults. Social capital as measured by less social participation (*AOR* = 1.55, 95% *CI*: 1.06–2.27), less cohesion (*AOR* = 0.63, 95% *CI*: 0.42–0.94), and less reciprocity (*AOR* =1.77, 95% *CI*: 1.17–2.68) were linked to self-rated health among older people with health conditions. While social capital regarding less social support (*AOR* = 2.15, 95% *CI*: 1.39–3.33) was related to self-rated health among older people without health conditions. We observed the interacting effect of social capital in the CART model, an implication that much focus should be geared toward vulnerable subgroups, especially depressed and lonely older people, as they have low reciprocity and little cohesion.

**Conclusion:**

This work demonstrates that social capital may be relevant in devising programs and measures to improve self-rated health among community-dwelling older adults with comorbidity.

## Introduction

The increasing aging population across the globe has compelled many nations to pay more attention to the health and well-being of older people (1). Empirical studies have concluded that self-rated health (hereafter referred to as SRH), a comprehensive concept and one of the subjective indicators of health (2, 3), is relevant in documenting the current status of health and forecasting health-related events likely to occur among older adults (4). Furthermore, previous investigations found that poor SRH is an important predictor of developing disability, morbidity, and mortality among older people (5, 6). Meanwhile, SRH is not only helpful in designing and implementing community health promotion and disease prevention programs but also in providing evidence on adult care (7).

Social capital is commonly referred to as public resources accessible and available to individuals through social relations and community engagement (8). In practice, several dimensions, such as social support, social connection, trust, cohesion, reciprocity, and social participation, were used to measure social capital (2, 9, 10). Previous studies have documented that social capital may be particularly beneficial for older people due to their degeneration in health and the increasing need for care and support in later life (1, 2). In recent decades, studies have explored the relationship between social capital and various health outcomes of older people (11–13), especially for SRH (14–17). Notably, such studies implicated social capital as a modifiable social resource that can be used in promoting SRH among older people in their later life (18–20). For instance, positive impacts of social capital (i.e., trust, civic participation and reciprocity, social support, and reciprocity) on SRH were observed among older people (9, 10, 21–23). In addition, the association between social capital and successful aging through the evaluation of subjective health and well-being has been documented (13, 24).

Although much research has confirmed the association of social capital with SRH among common older populations (2, 15, 25, 26). However, less is known about whether the association varies among older people with and without health conditions. Prior studies have proved that older individuals with comorbidities are vulnerable groups who have different perceptions of health which might impact their SRH (27, 28). Therefore, when designing programs or activities from the perspective of social capital to promote SRH among older people, it is necessary to examine such an association.

Furthermore, current studies exploring factors (such as, demographic, socio-economic, and social capital) individually associated with SRH are useful. Yet, how these factors together are associated with SRH is unclear. More comprehensive and sophisticated approaches are warranted to examine how these factors co-exist or interact with SRH (8, 17). Knowledge about these interactions will reveal potential mechanisms by which social capital is linked to SRH and guide more targeted and precise prevention programs in public health policy and clinical care practice. Given this, we employed a classification and regression tree (CART) model, which is a novel and sophisticated nonparametric approach and can be used to explore complex combinations or interactions among variables (29, 30).

To close these research gaps, we here aimed to achieve two main objectives. First, we aimed to examine the relationship between social capital and SRH and whether the association varies among older people with and without health conditions. Then, we explored the potential interactive effects of social capital and relevant factors on SRH among community-dwelling older adults.

## Materials and methods

### Study design and data collection

To obtain a representative sample, a multi-stage stratified cluster random sampling method was applied to recruit study participants. Following the local household registration system, individuals aged ≥60 years were ascertained. With the help and coordination of local community workers, each subject was personally visited at their home and engaged in a face-to-face interview by skilled and trained graduate students from the Anhui Medical University through a structured and interviewer-administered questionnaire. Before commencing the interviews, a verbal understanding of the purposes and procedures of the study and written informed consent were obtained. Initially, a total of 1,935 participants were interviewed, of whom 1,810 questionnaires were valid with a response rate of 93.5%. Details about our study design and sampling can also be found elsewhere (31–33).

### Measures

#### Self-rated health

Here, we considered SRH as the dependent variable, which was assessed with one general question. Typically, SRH, as a subjective indicator of health, was measured with a single item, which was reliable and valid based on findings from previous studies (1, 2, 34). In this study, participants were asked, “How do you rate your general health?” with responses ranging from very poor, poor, fair, good, and very good. For data analysis, we dichotomized the original responses into a binary variable: good health (0 = good, or very good) and poor health (1 = very poor, poor, or fair), which was similar to prior studies that investigated the relationship between social capital and SRH (3, 19, 35).

#### Social capital

Social capital, the main independent variable, was measured by a questionnaire containing six dimensions (social participation, social support, social connection, trust, cohesion, and reciprocity) and 22 items. For each domain of social capital, responses to the items were summed to generate an overall score. Notably, a higher score indicated a better social capital status. For data analysis, we dichotomized the scores of each dimension of social capital into two categories by taking the median value as the cut-off (36), such as social participation: high (≥6) and low (<6), social support: high (≥13) and low (<13), social connection: high (≥12) and low (<12), trust: high (≥13) and low (<13), cohesion: high (≥20) and low (<20), and reciprocity: high (≥11) and low (<11). Cronbach's α of the social capital questionnaire was 0.919. The details of the measurement have been previously published (31–33).

#### Other variables

Data on other variables, such as age (years), gender (male and female), body mass index (BMI; kg/m^2^), living status (not living alone and living alone), residence (urban and rural), marital status (married/cohabited and single), education (primary school and below, junior school, and high school and above), smoking status (non-smoking, former smoking, and smoking), drinking status (non-drinking, former drinking, and drinking) were collected. In addition, health-related variables, such as functional ability, depression, and loneliness were measured. In this study, a composite Activities of Daily Living (ADL)/Instrumental Activities of Daily Living (IADL) scale containing of 14 items was utilized to assess functional ability, the 16-item Zung Self-Rating Depression Scale (SDS) was employed to measure the depressive symptoms, and a single item to measure how the participants feel about the sense of loneliness. A more description of our measurement tools has been published elsewhere (31–33).

To identify respondents with health conditions, each participant was asked to provide answers to whether they had a clinical diagnosis of the following conditions or impairments: high blood pressure, diabetes, heart disease (coronary or valve disease), hyperlipidemia, angina, chronic bronchitis (chronic obstructive pulmonary disease [COPD]/emphysema), cerebral infarction (stroke), coronary heart disease (CHD), cataract, arthritis, cancer, and liver or kidney-related diseases. Eventually, 1,280 participants classified the group with health conditions if they reported at least one kind of the above-mentioned conditions (27). Accordingly, 530 participants were grouped as having no health conditions.

### Statistical analysis

Initially, the differences between good and poor SRH were evaluated by the chi-square test.

Second, to express our results, three binary logistic regression models with the odds ratio (*OR*) and adjusted odds ratio (*AOR*) and associated 95% confidence intervals (95% *CI*), namely, model 1, model 2, and model 3, were fitted separately. Based on previous studies (1, 37–39), we took some variables, such as age, gender, BMI, residence, living status, marital status, education, smoking status, and drinking status as potential covariates.

After this, we fitted a classification and regression tree (CART) model, with statistically significant variables in the unadjusted logistic regression model included, to examine the combined effect of social capital on SRH. In this model, SRH was categorized into subgroups by the most explanatory independent variables. Any possible interaction and combination with all social capital dimensions and other variables could generate these subgroups in the node. More detailed information about the CART model has been described in detail in our previous articles (31–33).

All statistical analyses were conducted using the Statistical Package for the Social Sciences, version 23.0 (SPSS Inc., Chicago, IL, USA). A two-sided level of 0.05 was considered significant.

## Results

### Results of descriptive analysis

Table 1 shows that over half of the sample was over 70 years old. As seen, about 72.3% (1,308/1,810) of the subjects reported poor SRH. Among them, 24.4% of the participants were aged between 65 and 69 years, and 58.8% of the respondents were female older adults. About 85.8% of the respondents were not living alone, 56.1% of them were residing in rural areas, 76.8% of them were married or cohabited, and 75.2% of the participants attended up to primary school. Notably, 78.4 and 83.6% of the subjects were non-smoking and non-drinking, respectively. More than half of the participants reported having a robust functional ability, and not being lonely while being depressed. Most of the participants with poor SRH reported a higher level of social capital concerning social participation, social connection, trust, and cohesion, but a lower level of social capital concerning social support and reciprocity.

**Table 1:** Characteristics of participants stratified by self-rated health (SRH) status (*N* = 1,810).

**Variables**	**Total** **(*N =* 1,810)**	**Phi and Cramer's V**	**Self-rated health**		**χ^2^**	***p*-value**
			**Poor** **(*N =* 1,308)**	**Good** **(*N =* 502)**		
**Age (years)**		0.124			27.905	<0.001
60–64	399 (22.0)		251 (19.2)	148 (29.5)		
65–69	424 (23.4)		319 (24.4)	105 (20.9)		
70–74	421 (23.3)		302 (23.1)	119 (23.7)		
75–79	282 (15.6)		225 (17.2)	57 (11.4)		
≥ 80	284 (15.7)		211 (16.1)	73 (14.5)		
**Sex**		0.044			3.431	0.064
Male	770 (42.5)		539 (41.2)	231 (46.0)		
Female	1,040 (57.5)		769 (58.8)	271 (54.0)		
**BMI (kg/m** ^**2**^ **)**		0.075			10.233	0.017
<18.5	189 (10.4)		148 (11.3)	41 (8.2)		
18.5–22.9	825 (45.6)		581 (44.4)	244 (48.6)		
23.0–27.4	644 (35.6)		457 (34.9)	187 (37.3)		
≥27.5	152 (8.4)		122 (9.3)	30 (6.0)		
**Living status**		0.052				
Not living alone	1,567 (86.6)		1,118 (85.5)	449 (89.4)	4.915	0.027
Living alone	243 (13.4)		190 (14.5)	53 (10.6)		
**Residence**		0.116			24.52	<0.001
Urban	801 (44.3)		532 (40.7)	269 (53.6)		
Rural	1,009 (55.7)		776 (59.3)	233 (46.4)		
**Marital status**		0.024			1.051	0.305
Married/cohabited	1,402 (77.5)		1,005 (76.8)	397 (79.1)		
Single	408 (22.5)		303 (23.2)	105 (20.9)		
**Education**		0.151			41.177	<0.001
Primary school and below	1,291 (71.3)		983 (75.2)	308 (61.4)		
Junior school	291 (16.1)		196 (15.0)	95 (18.9)		
High school and above	228 (12.6)		129 (9.9)	99 (19.7)		
**Smoking status**		0.014			0.367	0.832
Non-smoking	1,412 (78.0)		1,025 (78.4)	387 (77.1)		
Former smoking	99 (5.5)		71 (5.4)	28 (5.6)		
Smoking	299 (16.5)		212 (16.2)	87 (17.3)		
**Drinking status**		0.081			12.018	0.002
Non-drinking	1,484 (82.0)		1,094 (83.6)	390 (77.7)		
Former drinking	70 (3.9)		52 (4.0)	18 (3.6)		
Drinking	256 (14.1)		162 (12.4)	94 (18.7)		
**Number of disease**		0.421			321.185	<0.001
0	530 (29.3)		236 (18.0)	294 (58.6)		
1	714 (39.4)		556 (42.5)	158 (31.5)		
2	347 (19.2)		304 (23.2)	43 (8.6)		
>2	219 (12.1)		212 (16.2)	7 (1.4)		
**Functional ability**		0.189			64.586	<0.001
Robust	1,032 (57.0)		670 (51.2)	362 (72.1)		
Limited	778 (43.0)		638 (48.8)	140 (27.9)		
**Depression**		0.361			235.616	<0.001
Normal	890 (49.2)		497 (38.0)	393 (78.3)		
Depressed	920 (50.8)		811 (62.0)	109 (21.7)		
**Loneliness**		0.193			67.386	<0.001
No	1,110 (61.3)		726 (55.5)	384 (76.5)		
Yes	700 (38.7)		582 (44.5)	118 (23.5)		
**Social participation**		0.144			37.617	<0.001
High	1,043 (57.6)		696 (53.2)	347 (69.1)		
Low	767 (42.4)		612 (46.8)	155 (30.9)		
**Social support**		0.212			81.025	<0.001
High	906 (50.1)		569 (43.5)	337 (67.1)		
Low	904 (49.9)		739 (56.5)	165 (32.9)		
**Social connection**		0.142			36.332	<0.001
High	1,279 (70.7)		872 (66.7)	407 (81.1)		
Low	531 (29.3)		436 (33.3)	95 (18.9)		
**Trust**		0.200			72.279	<0.001
High	1,023 (56.5)		659 (50.4)	364 (72.5)		
Low	787 (43.5)		649 (49.6)	138 (27.5)		
**Cohesion**		0.169			51.403	<0.001
High	1,078 (59.6)		712 (54.4)	366 (72.9)		
Low	732 (40.4)		596 (45.6)	136 (27.1)		
**Reciprocity**		0.245			108.235	<0.001
High	978 (54.0)		608 (46.5)	370 (73.7)		
Low	832 (46.0)		700 (53.5)	132 (26.3)		

Among respondents who reported good and poor SRH, there were differences regarding age, body mass index, living status, residence, education, drinking status, number of diseases, functional ability, depression status, loneliness, and social capital dimensions.

### Results of logistic regression models

We fitted three logistic regression models (Table 2). In model 1 (the total subjects), two social capital dimensions were observed statistically associated with SRH, suggesting the adjusted odds ratio (*AOR*) of having poor SRH was shown to be 1.57 times (95% *CI*: 1.21–2.04) and 1.73 times (95% *CI*: 1.29–2.31) more likely for people with a lower social capital concerning social support and reciprocity, respectively.

**Table 2:** Results of the odds ratio (*OR*) for developing poor self-rated health.

	**Model 1**		**Model 2**		**Model 3**	
	**OR (95% CI)**	**AOR (95% CI)**	**OR (95% CI)**	**AOR** **(95% CI)**	**OR (95% CI)**	**AOR** **(95% CI)**
**Age (years)**						
60–64	REF.	REF.	REF.	REF.	REF.	REF.
65–69	1.79 (1.33–2.42)^***^	1.93 (1.37–2.72)^***^	1.92 (1.23–2.99)^**^	2.23 (1.35–3.68)^**^	1.07 (0.64–1.78)	1.12 (0.63–2.00)
70–74	1.50 (1.12–2.01)^**^	1.60 (1.14–2.24)^**^	1.41 (0.92–2.15)	1.66 (1.02–2.69)^*^	1.28 (0.79–2.07)	1.19 (0.67–2.10)
75–79	2.33 (1.63–3.32)^***^	2.31 (1.54–3.48)^***^	1.95 (1.19–3.21)^**^	2.25 (1.28–3.97)^**^	1.80 (0.99–3.28)	1.55 (0.77–3.14)
≥80	1.70 (1.22–2.38)^**^	1.38 (0.92–2.09)	1.33 (0.83–2.15)	1.27 (0.71–2.26)	2.13 (1.25–3.63)^**^	1.51 (0.75–3.04)
**Sex**						
Male	REF.	REF.	REF.	REF.	REF.	REF.
Female	1.22 (0.99–1.50)	1.10 (0.81–1.49)	1.30 (0.96–1.75)	1.27 (0.84–1.94)	0.99 (0.70–1.40)	0.92 (0.55–1.56)
**BMI (kg/m** ^**2**^ **)**						
18.5–22.9	REF.	REF.	REF.	REF.	REF.	REF.
<18.5	1.52 (1.04–2.21)^*^	1.33 (0.87–2.02)	1.89 (0.98–3.65)	1.67 (0.83–3.40)	1.94 (1.14–3.30)^*^	1.59 (0.85–2.97)
23.0–27.4	1.03 (0.82–1.29)	1.14 (0.88–1.48)	0.83 (0.60–1.14)	0.87 (0.61–1.24)	1.05 (0.71–1.56)	1.35 (0.86–2.11)
≥ 27.5	1.71 (1.11–2.62)^*^	1.78 (1.10–2.87)^*^	1.37 (0.76–2.46)	1.33 (0.69–2.54)	1.52 (0.72–3.20)	1.95 (0.83–4.54)
**Living status**						
Not living alone	REF.	REF.	REF.	REF.	REF.	REF.
Living alone	1.44 (1.04–1.99)^*^	1.29 (0.83–1.99)	1.67 (1.02–2.73)^*^	1.89 (1.01–3.54)^*^	1.06 (0.62–1.82)	0.76 (0.36–1.59)
**Residence**						
Urban	REF.	REF.	REF.	REF.	REF.	REF.
Rural	1.68 (1.37–2.07)^***^	1.19 (0.92–1.54)	1.65 (1.22–2.22)^**^	1.11 (0.78–1.59)	2.08 (1.47–2.96)^***^	1.51 (0.96–2.39)
**Marital status**						
Married/cohabited	REF.	REF.	REF.	REF.	REF.	REF.
Single	1.14 (0.89–1.46)	0.70 (0.49–1.00)^*^	0.99 (0.70–1.40)	0.52 (0.32–0.84)^**^	1.23 (0.81–1.88)	1.10 (0.61–2.01)
**Education**						
High school and above	REF.	REF.	REF.	REF.	REF.	REF.
Primary school and below	2.45 (1.83–3.28)^***^	1.21 (0.84–1.75)	2.54 (1.70–3.81)^***^	1.20 (0.74–1.97)	2.31 (1.37–3.88)^**^	1.29 (0.67–2.47)
Junior school	1.58 (1.11–2.27)^*^	1.29 (0.86–1.93)	1.64 (0.99–2.71)	1.36 (0.78–2.36)	1.53 (0.81–2.88)	1.24 (0.60–2.54)
**Smoking status**						
Non–smoking	REF.	REF.	REF.	REF.	REF.	REF.
Former smoking	0.96 (0.61–1.51)	1.35 (0.76–2.39)	0.71 (0.40–1.23)	1.19 (0.59–2.42)	0.50 (0.16–1.63)	0.49 (0.12–1.96)
Smoking	0.92 (0.70–1.21)	1.21 (0.83–1.77)	1.09 (0.70–1.68)	1.43 (0.82–2.51)	1.13 (0.74–1.72)	1.25 (0.69–2.27)
**Drinking status**						
Non-drinking	REF.	REF.	REF.	REF.	REF.	REF.
Former drinking	1.03 (0.60–1.78)	0.97 (0.50–1.89)	0.82 (0.42–1.61)	0.83 (0.36–1.89)	0.49 (0.12–1.91)	0.57 (0.12–2.65)
Drinking	0.61 (0.46–0.81)^***^	0.62 (0.43–0.88)^**^	0.73 (0.48–1.11)	0.74 (0.44–1.24)	0.64 (0.41–1.01)	0.53 (0.30–0.94)^*^
**Functional ability**						
Robust	REF.	REF.	REF.	REF.	REF.	REF.
Limited	2.46 (1.97–3.08)^***^	1.71 (1.29–2.27)^***^	2.27 (1.65–3.11)^***^	1.58 (1.07–2.34)^*^	1.93 (1.34–2.80)^***^	1.35 (0.82–2.22)
**Depression**						
Normal	REF.	REF.	REF.	REF.	REF.	REF.
Depressed	5.88 (4.63–7.48)^***^	3.75 (2.84–4.94)^***^	5.88 (4.16–8.30)^***^	3.78 (2.55–5.62)^***^	4.45 (3.04–6.51)^***^	2.93 (1.84–4.66)^***^
**Loneliness**						
No	REF.	REF.	REF.	REF.	REF.	REF.
Yes	2.61 (2.07–3.29)^***^	1.59 (1.21–2.09)^**^	2.51 (1.80–3.50)^***^	1.58 (1.07–2.32)^*^	2.06 (1.40–3.01)^***^	1.40 (0.88–2.22)
**Social participation**						
High	REF.	REF.	REF.	REF.	REF.	REF.
Low	1.97 (1.58–2.45)^***^	1.13 (0.87–1.47)	2.40 (1.73–3.33)^***^	1.55 (1.06–2.27)^*^	1.69 (1.19–2.40)^**^	0.87 (0.56–1.35)
**Social support**						
High	REF.	REF.	REF.	REF.	REF.	REF.
Low	2.65 (2.14–3.29)^***^	1.57 (1.21–2.04)^**^	2.32 (1.71–3.17)^***^	1.39 (0.96–2.02)	3.32 (2.32–4.75)^***^	2.15 (1.39–3.33)^**^
**Social connection**						
High	REF.	REF.	REF.	REF.	REF.	REF.
Low	2.14 (1.67–2.75)^***^	0.92 (0.68–1.24)	2.15 (1.49–3.10)^***^	0.99 (0.64–1.53)	1.95 (1.30–2.91)^**^	0.81 (0.49–1.34)
**Trust**						
High	REF.	REF.	REF.	REF.	REF.	REF.
Low	2.60 (2.08–3.25)^***^	1.23 (0.91–1.65)	2.45 (1.77–3.37)^***^	1.29 (0.85–1.95)	2.42 (1.68–3.48)^***^	1.05 (0.64–1.75)
**Cohesion**						
High	REF.	REF.	REF.	REF.	REF.	REF.
Low	2.25 (1.80–2.82)^***^	0.85 (0.63–1.14)	1.74 (1.27–2.38)^**^	0.63 (0.42–0.94)^*^	2.66 (1.83–3.87)^***^	1.13 (0.68–1.89)
**Reciprocity**						
High	REF.	REF.	REF.	REF.	REF.	REF.
Low	3.23 (2.57–4.05)^***^	1.73 (1.29–2.31)^***^	3.08 (2.23–4.27)^***^	1.77 (1.17–2.68)^**^	2.92 (2.03–4.22)^***^	1.59 (0.98–2.57)

Model 1, Model 2, and Model 3 included 1,810, 1,280, and 530 participants, respectively.

OR, odds ratio; AOR, adjusted odds ratio; 95% CI, 95% confidence intervals.

* p < 0.05;

** p < 0.01; and

*** p < 0.001.

In model 2 (subjects with health conditions), three capital dimensions were observed to be statistically associated with SRH, indicating that the *AOR* of experiencing poor SRH was shown to be 1.55 times (95% *CI*: 1.06–2.27), 0.63 times (95% *CI*: 0.42–0.94), and 1.77 times (95% *CI*: 1.17–2.68) more likely for people with a lower social capital as to social participation, cohesion, and reciprocity, respectively.

In model 3 (subjects without health conditions), statistical significance remained was only found for one social capital dimension, showing the AOR of experiencing poor SRH was shown to be 2.15 times (95% *CI*: 1.39–3.33) more likely for people with a lower social capital regarding social support.

### Results of the CART model

**Figure 1:**
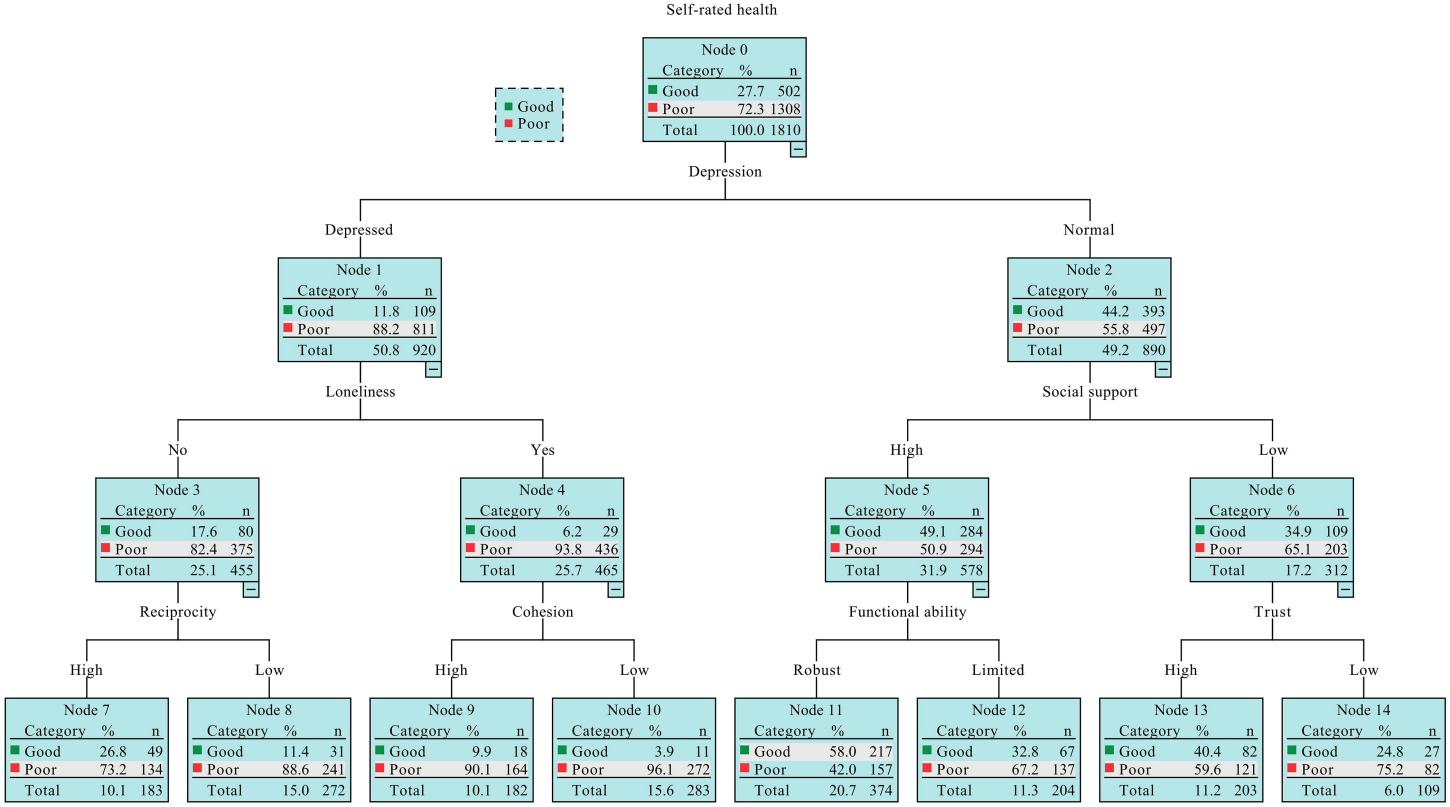
The classification and regression tree (CART) model output of the combinations of social capital factors and variables.

As shown in Figure 1 (the total subjects), depression, loneliness, social support, reciprocity, cohesion, functional ability, and trust were found to be linked to SRH, suggesting the interaction relationships between social capital and other variables were observed.

In particular, subjects who were depressed (Node 1) and lonely (Node 4), also reported a lower level of cohesion and were at the largest risk of experiencing poor SRH (Node 10). Meanwhile, those without depressive symptoms (Node 2), a high level of social support (Node 5) and a robust functional ability (Node 11) were least likely to experience poor SRH (Node 11). In addition, those who have less social support (Node 6) and less trust (Node 14) were inclined to suffer poor SRH when compared with those with a higher level of trust (Node 13). Similarly, those depressed respondents (Node 1) but not lonely (Node 3), who reported a lower level of reciprocity (Node 8) were more likely to suffer the poor SRH in comparison with those who reported a higher level of reciprocity (Node 7).

## Discussion

This study explored the relationship between social capital and SRH, with a focus on exploring whether the association varies with the presence of health conditions and how social capital, together with other variables, is linked to SRH among community-dwelling older adults in the Anhui province of China. We found that a few dimensions of social capital were associated with SRH and such associations varied by the presence of health conditions. Furthermore, potential mechanisms for how social capital is linked to SRH were reported. Specifically, older people with depression, loneliness, and functional ability limited, while reporting less social capital concerning social support, trust, cohesion, and reciprocity were prone to have poor SRH.

Our analyses indicated that, among the total participants, social capital in terms of social support and reciprocity, were positively associated with SRH, suggesting that older people who have more social support (such as, mental support and material support) and reciprocity (reciprocal willingness with their relatives, friends/neighbors, and strangers) are more likely to have better SRH, which is consistent with prior studies (10, 23). Similarly, such an association was also observed among older people without health conditions, which also suggested that high-level social support was a protective factor against poor SRH. While this association altered among older people with health conditions. In other words, social capital regarding social participation and reciprocity was positively connected with SRH, indicating the protective role of social participation and reciprocity for these communities. Though such protective roles were also documented in other studies (17, 40, 41), unlike these studies, our study paid special attention to these vulnerable groups, which validated the association of social capital with SRH is moderated by the presence of health conditions. In contrast to prior findings, which found the benefit of cohesion to SRH (42), our results reported that cohesion was negatively connected with SRH. A possible reason may be that older people with health problems are less willing to communicate or interact with others, thereby leading to less attachment to the surrounding community (43), which also emphasizes that more research is needed in the future. More importantly, the variations also emphasize that different measures concerning social capital to decrease the development of poor SRH should be well designed.

Nevertheless, based on our analyses, social connection, one of the six social capital indicators, was found to have no association with SRH, which was different from a prior study that reported an existing association of age peers with better SRH (44). This may be explained by the different items used to evaluate the social connection in the two studies. For example, in our study, 3 items (denoting the frequency of the older people getting in touch with their children, relatives, friends, or neighbors) were used to assess social connection, whereas that study used one item (indicating their agreement with the question about how they feel connected to their peers) to assess the association. This difference may imply that connection with peer groups is more crucial than connection with other individuals among older people. Of note, most of the previous research evaluated social capital using different indicators or dimensions. Therefore, findings on the role of social capital on SRH among older people are inconclusive, partly due to a lack of widely accepted definition and measurement of social capital (1).

In this work, we found a joint effect of social capital on SRH using a CART model. Other than the aforementioned social capital dimensions, such as social participation and trust (17, 40, 41, 45), social support (10), cohesion (42), and reciprocity (23), were found to be associated with SRH. Elsewhere, a study revealed that depression (46), loneliness (47), and functional ability (48), were associated with SRH among older adults. However, based on the current knowledge, few studies have explored how the interaction between these variables impacts SRH among older people. In the present study, depression was revealed to be the most important factor linked to SRH. Low levels of social capital, such as cohesion and depression with loneliness denoted the highest rate of developing poor SRH. Similarly, the combination of a low level of social capital (social support, reciprocity, and trust), loneliness, and limited functional ability suggests a greater possibility of the occurrence of poor SRH.

Our findings on the joint effects of social capital and other factors on SRH, not only, can be utilized to identify which subsets of elders are most liable to exhibit SRH. However, elucidating the potential mechanisms by which social capital is linked to SRH contributes to developing tailored and accurate measures to improve SRH among community-dwelling older adults. Specifically, special attention should be paid to those older people with health problems who are depressed and have functional ability limitations, also had a lower social capital, such as reciprocity. In addition, older adults without health conditions are encouraged to maintain good mental health, robust functional ability, and powerful social support from their children, relatives, friends, or community members to maximize the chance of having good SRH. More importantly, for general advanced age communities, to have good SRH, multiple programs or initiatives containing how to prevent depressive symptoms, loneliness, and functional limitation and cultivate the shape of social support, reciprocal, and trusting relationship with others, and cohesion in the community should be well designed in daily life. However, in addition to the above-mentioned significance and importance of the CART model results, some cautions should be made, as we described, this model is nonparametric, it is best to refer to the explanation together with another regression model when interpreting the result.

The present study has some major strengths. First, we utilized a valid and reliable scale, including six items to determine individual social capital in China. Hence, our work may promote the development of social capital in health research. Second, methodologically, we employed a sophisticated and comprehensive nonparametric CART model, which, together with results obtained by the logistic regression model, allowed us to explore the interplay of multiple variables and better explore the relationship between social capital and SRH. More importantly, we explored the interactive association between social capital and SRH, which further throws light on depicting potential mechanisms behind this association.

However, a few limitations should be acknowledged in our study. First, this was a cross-sectional study, making it hard to determine the causal association between social capital and SRH. Second, the data analyzed were collected by self-report, which could be associated with a risk of recall bias due to false or inaccurate responses from the participants. Furthermore, the social capital data analyzed in this study were only measured at an individual level, we did not include community-level social capital, and hence a study that incorporates community-level social capital may better appreciate the role of social capital theory in the future.

## Conclusions

This study provides evidence on the association between social capital and SRH, which shows that the relationship between social capital and SRH varies by the presence of health conditions among community-dwelling older people. Additionally, to better maintain SRH, much focus should be devoted to older people with health conditions and less reciprocity and social participation.

## Data availability statement

The raw data supporting the conclusions of this article will be made available by the authors, without undue reservation.

## Ethics statement

The studies involving human participants were reviewed and approved by Biomedical Ethics Committee, Anhui Medical University (No. 20150297). The patients/participants provided their written informed consent to participate in this study.

## Author contributions

ZB and JY participated in the survey, the data analysis, and writing of the article. ZW, WC, and CC contributed to the data collection. ZH and RC participated in the design of the study, contributed to quality control and data processing, and revised the manuscript. All authors have read and approved the final version.

## Funding

This work was supported by the National Natural Science Foundation of China (Nos. 71874002 and 71573002), the Special Research Project in Science and Technology Department of Anhui Province (No. 202106f01050045), the Key Project of Social Science in Education Department of Anhui Province (No. SK2021A0164), Research fund of Anhui Medical University (No. 2021xkjT049), and Open Program of Health Policy Research Center of Anhui Medical University (No. 2022wszc19). The organization had no role in the research design, data collection, data analysis, manuscript writing, and submission.

## Conflict of interest

The authors declare that the research was conducted in the absence of any commercial or financial relationships that could be construed as a potential conflict of interest.

## Publisher's note

All claims expressed in this article are solely those of the authors and do not necessarily represent those of their affiliated organizations, or those of the publisher, the editors and the reviewers. Any product that may be evaluated in this article, or claim that may be made by its manufacturer, is not guaranteed or endorsed by the publisher.
